# Stage-Based Model of User Engagement Patterns in an Online Health Community for Cardiovascular Disease Management: Qualitative Interview Study

**DOI:** 10.2196/90645

**Published:** 2026-10-05

**Authors:** Monisola Jayeoba, Yuyang Yang, Jingzhi Yu, Maia Jacobs

**Affiliations:** 1 Department of Communication Studies, School of Communication, Northwestern University, Evanston, IL, United States; 2 Department of Computer Science, McCormick School of Engineering and Applied Science, Northwestern University, Mudd Building, 3rd FL, 2233 Tech Dr, Evanston, IL, 60208, United States, 1 (847) 491-3500; 3 Department of Preventive Medicine, Feinberg School of Medicine, Northwestern University, Evanston, IL, United States

**Keywords:** online health communities, user engagement, user-centered design, disease management, American Heart Association

## Abstract

**Background:**

Online health communities (OHCs) provide vital peer support and health information to individuals managing chronic conditions. However, sustained user engagement remains challenging, with many users reducing activity over time despite the ongoing benefits these platforms offer. While some individuals may improve or become more knowledgeable, sustained engagement remains critical because ongoing participation fosters trust, peer support, and continuous access to evolving health information that may persist beyond initial recovery or learning. Hence, it is important to consider how community needs evolve as users’ health needs and information-seeking behaviors change to inform how we support users through different stages of their health and participation journeys.

**Objective:**

The aim of the study is to examine user engagement in a large OHC, identifying perceived stage-based behaviors, motivation, barriers, and design opportunities that could facilitate progression between stages and enhance long-term participation.

**Methods:**

We conducted semistructured interviews with 19 members of the American Heart Association Support Network Community. Participants were patients or survivors managing various cardiovascular diseases. Using narrative thematic analysis, we examined users’ perceived engagement motivation, behavior, challenges, and design opportunities across different stages of their community involvement.

**Results:**

This study highlighted 4 distinct engagement stages: discovery (crisis-driven initial engagement), exploration (navigation and orientation), commitment (active engagement and information management), and integration (sustained engagement and mentorship). Key barriers included information architecture complexity, concerns about misinformation, limited support for role transitions, and decreased participation as health management improved. Participants identified opportunities through which OHCs could increase long-term engagement, including adaptive recommendation systems, health information literacy programs, structured role transition support, and alternative engagement modalities, such as synchronous interactions and health tracking tools.

**Conclusions:**

User engagement in OHCs is dynamic and evolves with changes in health status, knowledge, and personal circumstances. Supporting sustained engagement requires stage-appropriate interventions, including personalized content delivery, health information literacy education, structured pathways for role transitions, and diversified engagement options. These findings provide actionable insights for designing OHCs that better support users throughout their health journey.

## Introduction

Online health communities (OHCs) have been widely adopted as pivotal resources in digital health, offering internet-based platforms where individuals can access peer support, share experiences, and seek health-related information [1-5]. These communities are particularly valuable for individuals managing both acute health events and chronic conditions [6] by facilitating social support, including informational support, emotional support, and companionship to geographically dispersed users [7-12]. Such support has been linked to positive health outcomes, including healthy lifestyles [13,14], patient empowerment through enhanced health knowledge [15,16], reduced uncertainty around diagnosis and treatment [16], improved attitudes toward chronic illness [11], and reduced rural-urban health disparities [9].

A critical aspect often overlooked is that user engagement in OHCs is not static but evolves with changes in users’ health status, knowledge, motivation, and personal circumstances. Despite the recognized benefits, many users only engage for a short period of time [17], and long-term engagement in OHCs is important to create sustained value for both individuals and the collectives in these communities. For individuals, ongoing participation fosters trust, provides continuous access to reliable information, and builds supportive relationships that can improve health outcomes and emotional well-being. For the community, consistent engagement ensures the exchange of diverse experiences, strengthens peer-to-peer support networks, and maintains a dynamic knowledge base that evolves with members’ needs [18,19]. Together, these factors transform the community into a trusted, resilient space where health behaviors and outcomes can be positively influenced over time.

User engagement—defined as the behavioral and psychological involvement of users, characterized by active participation, willingness to contribute, and investment of time and energy—is crucial for the ongoing success and sustainability of these communities [20-22]. Existing studies have highlighted several facilitating drivers of engagement. For example, research has identified that perceived usefulness and patient satisfaction are influenced by social support, information quality, and service quality [23,24]. Another study highlighted the importance of meeting users’ needs for autonomy, competence, and relatedness through mechanisms that promote choice, knowledge-sharing, and social connection [5]. Most recently, Cao et al [25] highlighted self-efficacy and outcome expectations, system quality, information quality, and social interaction ties.

While prior studies have identified several facilitating factors, engagement and active participation in OHCs often remain difficult to maintain over time; research consistently shows that many users, after initially participating actively, gradually reduce their activity or discontinue engagement altogether [23,26]. To support sustained engagement in OHCs, it is important to understand the perspectives of members at various time points in their health and information-seeking journeys, and how they might be better supported through these different stages. Hence, this study aims to examine engagement patterns within a large OHC, identify users’ motivations and barriers at each stage, and understand the needs that facilitate progression between stages. Based on these insights, we propose design strategies to support sustained participation and enhance the long-term value of OHCs.

To achieve this research aim, we conducted a qualitative study of 19 members of the American Heart Association (AHA) Support Network Community (SNC), using semistructured interviews and thematic narrative analysis to uncover perceived engagement patterns, from initial entry to more sustained involvement, and how design interventions might better support users at different points in their participation. Our findings reveal a nonlinear stage-based model of perceived engagement patterns that unfold in 4 stages, namely, discovery, exploration, commitment, and integration, and are shaped by mechanisms such as self-efficacy building, trust formation, and role transition. Each stage reflects distinct perceived behaviors, motivations, and challenges, offering actionable insights for improving user experience. These insights inform design opportunities, including adaptive personalization, health literacy interventions, and structured pathways for mentorship. Understanding these engagement trajectories is critical for designing evidence-based interventions that sustain long-term participation, peer connection, and knowledge exchange in OHCs, with the ultimate goal of improving patient outcomes in digital health contexts. The remainder of this paper is organized as follows: the Methods section details the study design, recruitment, and analysis approach; the Results section presents a stage-based model of engagement and the associated user behaviors and barriers; and the Discussion section interprets these findings in relation to existing literature and outlines design implications.

## Methods

### Study Setting

This qualitative study used semistructured interviews to explore the dynamic nature of user engagement in OHCs. A qualitative approach was chosen because it offers the flexibility to explore the depth and complexities of users’ attitudes, behaviors, and lived experiences, aspects that are difficult to capture through quantitative methods alone [27-29]. This approach enabled us to examine the engagement patterns that emerged, understand why users engaged in particular ways, and identify their evolving needs across different points in their health information-seeking and self-management journeys.

The study was conducted within the AHA SNC, a US-based OHC that provides social support and health education to individuals and their caregivers managing various forms of cardiovascular disease (CVD). The platform offers several key features typical of established OHCs, including waiting room support for new members, small interaction groups organized by predominant CVD types, discussion boards where members can ask questions and share knowledge, and moderated educational content and resources such as news and academic articles curated by community managers. At the time this study was conducted, the program managers reported that the AHA OHC had about 12,000 registered users.

Throughout the study, we used a participatory research approach, collaborating closely with community managers who serve as important stakeholders in how OHCs function. This collaboration ensured that our research instruments were contextually appropriate and that findings would be actionable for community improvement.

### Participant Recruitment

Participants were recruited through the AHA SNC. The research team developed a recruitment survey containing eligibility questions and baseline sociodemographic items. This survey was iteratively reviewed and revised with community managers to ensure relevance and appropriateness for the community context. Community managers then distributed the survey across the platform via community announcements and direct communication channels, inviting interested members to participate.

Survey completion was voluntary, and only individuals who completed the survey, indicated interest in participating, and met eligibility criteria were invited to the interview. Eligibility criteria were intentionally broad to capture diverse engagement experiences: (1) participants must be at least 18 years of age, and (2) participants must be current members of the AHA SNC, regardless of membership duration, engagement level, or specific cardiovascular condition.

Interviews were conducted in batches with concurrent data analysis, allowing emerging insights to inform subsequent interviews while maintaining consistency in core questions. An initial round of recruitment revealed demographic imbalances, particularly an overrepresentation of White participants, prompting targeted recruitment of underrepresented participants. Recruitment continued until data saturation was achieved [30], defined as the point at which new interviews provided existing insights and themes became well-established across the dataset. A total of 19 participants were interviewed for this study.

### Data Collection

We collected data through one-on-one semistructured interviews via Zoom (Zoom Video Communications) audio over 2 months, from mid-June to mid-August 2023. Each interview lasted approximately 1 hour. The lead author (M Jayeoba) conducted all interviews, using a semistructured protocol guide that allowed for flexible exploration of participant experiences while ensuring systematic coverage of key topics across all interviews [31]. The interview protocol explored initial engagement and engagement patterns, information exchange behaviors, role evolution, platform usability and navigation, unmet needs and barriers, and design preferences and suggestions (Multimedia Appendix 1).

Before each interview, participants were reminded of their rights, and the interviewer sought oral confirmation of consent in addition to the written consent previously obtained. Participants were informed that audio would be recorded for analysis purposes. All sessions were recorded and saved to Zoom Cloud Service, then professionally transcribed by Rev human transcription service to ensure accuracy.

Throughout data collection, M Jayeoba maintained detailed reflexive memos documenting observations, emerging patterns, methodological considerations, and questions for follow-up. These memos were discussed regularly with the research team, who provided feedback and suggestions for adapting the interview protocol as patterns emerged. This iterative approach allowed us to probe more deeply into unexpected themes while maintaining consistency in data collection. The study did not offer monetary compensation; however, the interviewer and community managers thanked the participants for their time and knowledge contributions.

### Data Analysis and Quality Measures

The interview transcripts and memos were imported into Microsoft Excel and MAXQDA (VERBI Software) for data analysis and management. Upon reviewing the interview transcripts, we identified discrepancies in role designation for 2 of 3 participants who identified as family or friend and formal caregiver in the recruitment survey. The 2 participants’ interview narratives described their own recent diagnoses and personal health journeys. One of the 2 participants explicitly stated joining the community after being diagnosed and described personal experiences consistent with those of patients. The second one mentioned that they initially joined the community as a caregiver but later received a personal diagnosis that prompted them to seek support for their own health. Since participants received no financial incentive, we believe these discrepancies likely stemmed from unintentional survey errors rather than misrepresentation. To preserve analytic coherence, these participants were reclassified as patients. Additionally, we excluded the data from the third participant who identified as a formal caregiver since we no longer had sufficient representation from this user group. Our final analytic sample conclusively includes 19 individuals managing cardiovascular conditions.

To analyze our data, we used thematic narrative analysis, a qualitative method that combines thematic analysis with narrative analysis [32]. Thematic analysis guided our coding and theme construction process [33,34], while narrative analysis was used to organize participants’ retrospective experiences with the AHA SNC into stages [32,35]. This blending reflects a methodological stance that meaning in chronic illness contexts is often best understood through patients’ storytelling [36], and applying multiple methods of systematic evaluation, such as thematic and narrative techniques, can increase interpretive validity and lead to richer, more accurate representations of lived experience [36,37]. Meanings were created inductively at a semantic level to capture community members’ stories and reflections. Despite this methodological flexibility, our thematic narrative analysis processes followed a systematic approach that was structured into 6 stages [33,34]. We initially performed data immersion, reflecting deeply on the data for familiarization, meticulously reviewing the transcripts, and verifying their accuracy and completeness against the audio recordings. Following this, we first developed preliminary codes from open coding of 5 transcripts using Microsoft Excel. These codes were refined through team discussions to resolve discrepancies and ensure coherence, and Microsoft Excel provided the flexibility for that. The final code structure was then transferred into MAXQDA for structured analysis of the remaining 14 transcripts, allowing for organized data management and code frequency review.

The participant data were not analyzed solely based on their current engagement stage, as their narratives spanned the breadth of their experiences since first discovering and joining the OHC. Instead, we clustered these diverse experiences and narrated them into progressive yet nonlinear stages of engagement. This approach acknowledges our reflection on the data, that participants do not always fit neatly into a single stage at a given moment, but rather exhibit behaviors and motivations that traverse multiple stages, offering a holistic understanding of their long-term engagement journey. Coding decisions evolved through continuous refinement until conceptual saturation was determined when no new themes emerged across successive interviews [30,38]. After the 19 transcripts were coded, the lead researcher (M Jayeoba) grouped the codes into potential themes, and all the authors discussed the themes to mitigate bias and establish congruence. The discussion led to collaborative definitions and labels of the themes that comprise the thematic map of the analysis.

After data analysis, we compiled a high-level report summarizing each theme, including representative participant quotes, and presented it to the AHA community managers in a virtual feedback session. This session was conducted to ensure that those responsible for managing the platform were informed by participants’ perspectives and could use the insights to guide decision-making and improve community engagement strategies. During the meeting, we invited open-ended reflections on the findings, where community managers noted that many of the findings aligned with their own observations, but we did not systematically analyze this feedback. While the community managers’ input did not influence our coding or themes, it provided an informal validation and practical relevance of our findings from a stakeholder perspective.

Finally, to enhance research quality, we used several strategies recommended for qualitative inquiry [39] (Checklist 1). We documented all analytical decisions and theme development processes. Multiple researchers were involved in data interpretation to provide diverse perspectives and challenge assumptions. Member checking occurred informally through our ongoing collaboration with community managers, who reviewed emerging themes and confirmed alignment with their observations.

### Ethical Considerations

The protocol for this study was jointly developed with the community and program managers of the AHA SNC to ensure compliance with community values and ethical standards. The institutional review board at Northwestern University reviewed the protocols and granted an exemption (STU00216828). Before data collection, all participants provided written informed consent via REDCap (Vanderbilt University Medical Center). Participants had opportunities to clarify ethical concerns before engaging in the study and were reminded of their rights at the start of each interview session. All data were anonymized with names and identifying details removed to ensure privacy compliance. Participants did not receive monetary compensation for their time, as the project was not funded. However, the researchers and program managers sincerely thanked the participants for their time and contributions.

## Results

### Overview

This section presents our analysis of user engagement patterns in an online cardiovascular health community through a stage-based model. Our study included 19 members of the AHA SNC who participated in the semistructured interviews. The sample included 10 female and 9 male participants, with participants identifying as Asian (n=1), Black or African American (n=1), Hispanic or Latino (n=1), White (n=15), and other (n=1) backgrounds. Most participants were highly educated, with 10 participants holding advanced degrees (MA, MS, MBA, PhD, MD, and JD), 5 having completed college degrees (BA or BS), and 4 having some college, technical degrees, or associate degrees. The sample was primarily older adults, with the age distribution showing concentration in the 65‐ to 74-year range, reflecting a mature cohort navigating the complexities of long-term health management. Participants reported diverse cardiovascular conditions, with many managing multiple diagnoses simultaneously. It is worth noting that all the study participants used the same OHC platform and information architecture, or set of features, within the platform, thereby contributing to the validity of our method and results. See Table 1 for detailed participants’ sociodemographic and health characteristics (N=19).

**Table 1.** Sociodemographic and health characteristics as reported by study participants (N=19).

Characteristics	Values
Sex, n (%)	
Female	10 (52.6)
Male	9 (47.4)
Race or ethnicity, n (%)	
Asian	1 (5.3)
Black or African American	1 (5.3)
Hispanic or Latino	1 (5.3)
White	15 (78.9)
Other	1 (5.3)
Age group (years), n (%)	
35‐39	1 (5.3)
40‐49	0 (0)
50‐59	1 (5.3)
60‐64	3 (15.8)
65‐69	5 (26.3)
70‐74	5 (26.3)
75‐79	3 (15.8)
80‐84	1 (5.3)
Education level, n (%)	
Advanced degree (MA, MS, MBA, PhD, MD, JD)	10 (52.6)
College degree (BA or BS)	5 (26.3)
Some college or technical degree or associate	4 (21.1)
Cardiovascular conditions, n (%)	
Atrial fibrillation	9 (47.4)
Cardiac arrest or sudden cardiac arrest	4 (21.1)
Heart disease or coronary artery disease	4 (21.1)
Tachycardia or supraventricular tachycardia	4 (21.1)
Heart failure	3 (15.8)
Heart valve disease—mitral valve	3 (15.8)
Peripheral arterial disease	3 (15.8)
Cardiomyopathy—ATTR-CM^a^	2 (10.5)
Heart attack or myocardial infarction	2 (10.5)
Heart valve disease—aortic valve	2 (10.5)
Stroke—ischemic	2 (10.5)
Number of concurrent CVD^b^ conditions, median (range)	2 (1‐7)
Total unique conditions represented, n (%)	11(57.9%)
Affiliation with AHA^c^ Community, n (%)	
Patient or survivor	19 (100)

a ATTR-CM: transthyretin amyloid cardiomyopathy.

b CVD: cardiovascular disease.

c AHA: American Heart Association.

Our analysis reveals how members progress through 4 distinct stages of engagement in the AHA SNC: discovery, exploration, commitment, and integration. The stages represent phenomenological or retrospective journey reconstructions rather than true time-series trajectories. At the discovery stage, members exhibit initial awareness with limited engagement, primarily focusing on orientation needs. During exploration, members begin trying out features and engaging in some interaction, and their need for connection becomes paramount. As members advance to the commitment stage, they demonstrate consistent participation and relationship building, often taking on supportive roles and seeking recognition for their contributions. Finally, in the integration stage, members achieve sustained but flexible participation, requiring support to maintain a healthy balance in their community engagement. We organized our findings into 2 broad categories: first, we identified stage-specific engagement patterns and the challenges affecting progression through each stage; second, we uncovered emerging opportunities to support members’ needs at each stage of their community involvement. See Textbox 1 for the categories and themes that showcase the perceived stage-specific engagement patterns and the design opportunities to facilitate long-term engagement across stages.

Textbox 1. **Categories and themes that showcase perceived stage-specific engagement patterns and the design opportunities to facilitate long-term engagement across stages.**

**Perceived stage-specific engagement patterns**

- Theme 1: discovery stage—crisis-driven initial engagement
- Theme 2: exploration stage—navigation and orientation
- Theme 3: commitment stage—active engagement and information management
- Theme 4: community integration stage—sustaining engagement long-term and forging community integration

**Design opportunities for facilitating long-term engagement**

- Theme 1: opportunity for adaptive recommendation system to improve stage-based personalization
- Theme 2: opportunity for patient education to optimize use practices
- Theme 3: facilitating and supporting role transitions within the community
- Theme 4: interest in alternative activities and modes of engagement

### Perceived Stage-Specific Engagement Patterns and the Challenges

#### Theme 1: Discovery Stage—Crisis-Driven Initial Engagement

##### Overview

The discovery stage emerges as a critical period for most participants, characterized by intense emotional support and informational need, often following “shocking” CVD diagnoses. Our analysis revealed that this stage encompasses 2 key dimensions: the immediate impact of diagnosis and recognition of information gaps.

##### Subtheme 1: Diagnosis as a Trigger Point for Immediate Engagement

The discovery stage typically begins at the point of diagnosis, triggering urgent information and emotional support and regulation needs. Participants described entering the community during moments of shock and uncertainty, motivated by the desire to connect with others who share similar experiences, as Participant 4 shared:

The reason that I was interested was that I was recently diagnosed with heart failure and pulmonary artery hypertension. Well, I thought that it would be helpful to me to be in communication with other people who had diagnoses like these ... So, it’s shocking in a way.

As illustrated by Participant 4’s quote, emotional distress often drove participants to seek social support and observational learning from others’ lived experiences. Consequently, users exhibited curiosity, often browsing and assessing the platform’s value, and sometimes asked questions to address their immediate concerns.

##### Subtheme 2: Information Gaps Between Clinical Care and Self-Management Needs

A primary motivator that emerged for considering community participation at this stage is self-efficacy building. Most participants recounted getting diagnosed, feeling very confused, and sought more practical advice than clinical instructions, highlighting gaps between medical guidance and everyday self-management. As Participant 5 shared:

... sometimes your doctor just doesn’t know. They know about your medication, your surgery ... And sometimes the lifestyle modifications for them are a little fuzzy. So, for example, they might tell somebody you should be on a low-sodium diet. But when it comes down to brass tacks for a person to ask, well how do I reduce sodium in my diet? What do I cook? That’s where the forum could be helpful because it’s people who’ve done it and have advice on what to buy and what to cook.

This sentiment was echoed by another participant, who noted:

*No, my doctor, they go in there quick, and they do an EKG and they say, “Oh, this is bad.” They don’t go into too much detail on how to live or what you need.*

[Participant 10]

Many participants discussed that while physicians effectively delivered diagnoses and medication protocols, they offered substantially limited guidance for practical lifestyle modifications, prompting members to turn to the community for actionable advice. The reliance on peer-shared strategies reflects self-efficacy development as users sought to build confidence in managing their condition through practical, experience-based knowledge from peers.

##### Subtheme 3: Challenges at the Discovery Stage

Despite strong motivations to engage, participants faced significant challenges during this initial phase, including information overload and health and technical literacy gaps, which strained emotional coping and reduced their ability to process content.

While having access to social support was highly appreciated, most participants reported being overwhelmed and initially encountering challenges with processing the volume of available health information on the platform during the early stages of their diagnosis. As Participant 7 highlighted:

I think what sometimes is [the reason] for low engagement is that, in the beginning of somebody’s diagnosis ... You’re so overwhelmed. I think it’s hard for people to find the time when they’re so overwhelmed to actually ask for help.

Similarly, health literacy gaps and jargon eroded self-efficacy and confidence, making users feel excluded. Users struggled with medical terminology while dealing with the emotional impact of their diagnosis. Participant 8 pointed out that the community often assumes a baseline level of health literacy among users, saying that:

I think in communicating from an advocacy group, a health information group, or any kind of expert group, that there is too much reliance on accepted jargon and too much presumption that everybody knows, for example, what chest pain means, let’s say.

Ultimately, these barriers create a challenging environment for meaningful engagement. Overwhelming content and jargon weaken self-efficacy, while a lack of clarity in communication limits observational learning and social support within the community.

### Theme 2: Exploration Stage—Navigation and Orientation

#### Overview

The exploration stage is marked by active information-seeking and relationship-building patterns. Users move from initial exposure to actively exploring features and resources. Participants in this stage made concrete efforts to understand the community’s structure and find their place. Engagement shifts from passive observation to intentional information-seeking, often leveraging site features to meet their informational and emotional needs. Participants reported low-to-moderate interactions (eg, asking questions or reading articles), but growing self-efficacy and curiosity drove their increased activity.

#### Subtheme 2.1: Active Information-Seeking Behavior

Participants described using platform tools (like the search bar) to locate relevant topics and validate peer insights, reflecting confidence-building and observational learning. For example, Participant 11 explained how they would search for others’ experiences and then ask their own questions:

I researched ... I did on the search box about things related to what I was experiencing and then I check other peer people, other peer asking questions, and I read what they were describing, their event ... And then after I read, I digest that a bit, and then I went back and asked questions.

This cyclical process of searching, reading, reflecting, and then asking questions demonstrates self-efficacy development through successful navigation, information retrieval, and learning.

Participants also emphasized the importance of timely responses, particularly from moderators, as a signal of the platform’s trustworthiness and motivation to continue engaging. Reflecting on early experiences, Participant 11 shared that:

I was amazed about how the moderators, they reply so soon ... So it’s a very interactive way. You just don’t have to wait for weeks till someone address. I thought that I’m going to be ignored, but that was not the case. So I was really very amazed that that was really real-time ...

This highlights how real-time interaction and prompt feedback can boost user satisfaction and self-efficacy during the exploration stage. When users saw their questions addressed almost immediately, it reinforced their trust in the community and motivated further participation.

#### Subtheme 2.2: Interest in Relationship-Building and Shared Understanding

Most participants stressed the importance of connecting with peers who shared similar health experiences, conditions, or circumstances (eg, age group). They sought emotional resonance and empathetic communication through those shared experiences to foster a sense of belonging in the community. As Participant 10 expressed:

I would like to communicate with other people, and there must be people out there that are in the same boat as me, especially at my age that have the same situation.

This desire for relatable connection suggests that relationship-building, particularly through condition-specific or demographic subgroups, is a crucial foundation for progressive, meaningful engagement. For example, Participant 10 initially sought context-specific information over emotional support, saying:

I was looking for people in my situation, not somebody else ... I don’t want sympathy, put it that way. And that’s what it seems to be, it seems to be mostly, “Oh yeah, I know you feel bad,” and stuff like that. But I don’t want that at the moment, I want information about my situation.

However, Participant 10’s subsequent comments revealed a nuanced need for connection within shared relational contexts:

If they could subdivide it up a little bit into the types of disease ... get the people that have the same thing and get them together somehow ... subdivided like that, and I would log into that. If somebody said, oh they’re going to have the same thing done as I did, an open heart [surgery] ... I would tell them flat out what I felt and what happened to me.

Participant 10’s perspective illustrates that establishing relevant peer relationships can foster reciprocity and knowledge exchange, increasing a user’s willingness to engage more when interactions are grounded in shared experience. While members like Participant 10 may have initially been focused on gathering information, providing opportunities to connect with peers who have similar health journeys made them more open to deeper, more supportive engagement. These relationships enhance immediate participation, enable shifts in engagement priorities, and empower users to transition into more active and supportive roles.

#### Subtheme 2.3: Challenges at the Exploration Stage

Despite their enthusiasm to explore the community, participants encountered challenges undermining their ability to effectively use the platform, most prominently, information architecture complexity, limited personalized content, and insufficient social ties.

##### Information Architecture Complexity

Some participants noted not feeling efficacious because the information structure on the community’s website was difficult to navigate, which undermined their confidence. New users, in particular, felt frustrated when trying to find content relevant to their needs. As one participant described:

Because I find that, even going to AHA’s website, if I don’t ask the question in a way it’s in your database, for example, I may not find it, or if I don’t use the right language, I may find a topic but not necessarily the answer to a question.

[Participant 8]

A few experienced members echoed these navigation difficulties. Even with moderator-provided resources and organized content, some users still had to take multiple steps or dig through layers of menus to locate specific information. Participant 3 recounted that often it was not straightforward to get to the exact support they needed:

... Often you can’t find what you need without taking several steps, you’re not going to find exactly the target without going through the outer concentric rings to get to the center of that target.

When met with confusing site structure and layout, some participants felt that finding the right information was a tedious process. This complexity eroded some users’ self-efficacy, discouraging some from deeper exploration.

##### Unmet Personalized Needs

Unmet personalized needs weakened users’ goal alignment, as users struggled to find content relevant to their conditions. For most participants who had initially joined the community with high curiosity-driven information-seeking intention, the lack of tailored content often led to further narrowing of their interactions. A few of the participants expressed dissatisfaction with generalized content after joining the community. Some participants attributed the lack of personalized content to reduced usefulness, which, consequently, they reported as a major influence on their reduced engagement in the community, as Participant 2 shared:

... for example, this week was about strokes, ... previous weeks have been about other heart-related issues ..., let’s say I made a mistake and I told them I had a stroke. Well, why am I not consistently receiving info related to strokes?

In addition to unmet personalized information needs, some participants also reported having limited emotional support needs that go beyond question-and-answer, which restricted social identity formation, leaving users feeling disconnected. Participant 2 expressed that:

You write into somebody. They give them advice, but there’s no dialogue among the people who are writing in, and one or two people might respond to the person who writes in about their distress, but there’s no ongoing support for them, and that’s what I was looking for.

When participants perceived their social support needs, that is, information and emotional support, the outcomes that most participants sought in OHCs were unmet; they reported having reduced platform use and exhibited selective engagement with the community. Specifically, they highlighted limited personalized content for information support and social ties for emotional support as challenges they encountered as they explored the community’s value. Put together, these barriers illustrate how platform design can either reinforce or erode mechanisms that are critical for progression.

### Theme 3: Commitment Stage—Active Engagement and Information Management

#### Overview

The commitment stage represents a mature phase of community participation defined by regular engagement. At this stage, members consistently participate and actively both seek and share information. Having overcome initial shock and orientation challenges, many participants felt more confident managing health information and interacting in the community; yet, they remained mindful of information quality and risks.

Participants at the commitment stage showed a strong dedication to building community relationships and social support networks. They viewed themselves as core community members invested in helping the community thrive. Notably, their engagement exhibited two key behavioral patterns: (1) taking responsibility for community norms and ensuring quality interactions and (2) providing support and mentorship in the community.

#### Subtheme 3.1: Taking Responsibility for the Community Norm and Interaction Quality

As members settled into this active engagement stage, they developed careful approaches to information exchange. They were highly aware of the potential pitfalls of online health information, expressing concern about inadvertently spreading misinformation or being misinformed themselves. This heightened risk awareness led to cautious, responsible practices in how they sought, shared, and evaluated content. Participants strove to avoid contributing anything that might mislead others. We observed this through their use of disclaimers, selective topic choices, and other strategies to mitigate misinformation.

For example, with the use of disclaimers, a participant sharing how they typically began their posts by clarifying that they are not health professionals, to emphasize the limits of their advice mentioned that:

*I’m not a medical doctor ... You always have to say [that]. So I’m comfortable engaging because I feel like I’m covering my butt enough by saying that.*

[Participant 7]

By prefacing their contributions with disclaimers, participants signaled that their advice was based on personal experience rather than professional expertise. These disclaimers appeared to help them manage concerns about overstepping or unintentionally misleading others, while still allowing them to participate in information sharing. In doing so, participants also reinforced community norms around cautious and responsible health information exchange.

Similarly, this study identified that as users became more deeply involved, they developed individual and community-wide strategies to ensure that information was trustworthy. Recognizing that much of the content shared in the community was opinion-based rather than expert-reviewed necessitated some users to critically assess the information they encountered.

As part of this process, some participants demonstrated increased self-efficacy by cross-referencing information with moderator-provided resources and endorsed links, such as medical journals or official health websites, as Participant 5 mentioned: *“*You just have to be aware that it is opinions and to go and look further in the resources put by the site or the moderator.”

In addition, some participants demonstrated trust formation through reliance on moderators for credible information and viewed moderated content as a key factor in maintaining trust within the community. They emphasized that the presence of moderators who actively validated content reassured them about the platform’s trustworthiness:

I would say excellent and outstanding trustworthiness, totally support that I can trust the sites. Especially because they’re moderated and the moderators they come and they answer. Their [moderator] comments are great, but they go above and beyond and put some links that are really, really good. And I normally went to those links and found them very helpful.

[Participant 5]

This trust in moderators reflects the value placed on structured content validation and a proactive stance toward mitigating misinformation risks and consuming reliable resources.

#### Subtheme 3.2: Providing Support and Mentorship in the Community

As members entered the commitment stage, many shifted from seeking help to actively helping others. They began to take on roles that enriched the community’s collaborative environment. Participants described deepening their interactions and seeking to mentor newer members by sharing personal experiences. They also expressed a growing sense of responsibility for the community’s vitality, seeing their own contributions as essential to keeping the community vibrant. Motivated by the social support they have received, some of our study participants at the commitment stage demonstrated relational reciprocity. At this stage, users reported transitioning from information seekers to information sharers, actively seeking ways to provide support to newer or peer members. However, we observed that user roles and engagement levels in OHCs are dynamic, with users transitioning from information seekers to providers, and vice versa, and sometimes exhibiting dual role dynamics, depending on several factors. Participant 7 exemplifies this fluidity, offering a nuanced case of shifting motivations and user behavior from caregiver to peer leader and being a patient-survivor. Participant 7 initially joined the community as a caregiver but later received a personal diagnosis that prompted them to seek support for their own health.

The participant described entering the community with a clear goal of seeking information and resources, particularly educational materials such as articles and videos. However, Participant 7 received recognition from the community managers, asking them to share experiences in a more recognizable format:

... I posted something, asked a question, or something. And then I got asked to write an article about how we were doing. And that’s how I got started. I probably first joined it looking for information. But right now, I was very active in the network part, and then I became a peer group leader. So, I guess it started out where I was looking for help. And now, looking to help.

This attention seemed to have primarily empowered Participant 7 to take on greater responsibilities and commit to the community. Over time, Participant 7’s role evolved as they moved from seeking support to providing support, which is indicative of the transition to the commitment stage.

Although Participant 7’s support role intensified, the participant recounted a subsequent diagnosis that introduced new personal health challenges and their need for support and information:

But also, in the past year, I got diagnosed with extremely high cholesterol, and I’m statin intolerant. So now, I’m back to looking back and trying to get information also ...

Beyond taking on support roles, commitment-stage members increasingly recognized their influence on others and felt accountable for the community’s well-being. They developed a sense of ownership in making sure that the platform remained a helpful, caring space. This heightened responsibility often fueled their long-term commitment. As Participant 7 illustrated with a personal anecdote:

... She posted, “I don’t think I can do this. I don’t know what to do.” So, it’s my responsibility, I feel, to check it every day and make sure there’s not somebody out there that’s needing a lifeline.

This level of dedication suggests that helping veteran members feel impactful is key to sustaining engagement. Communities can support these leaders through resources and recognition that enable them to continue their valuable work. For instance, the platform could introduce features like special notifications for urgent posts or distress signals, so committed members like Participant 7 can quickly identify and assist peers in crisis. Empowering users in this way helps maintain the community’s supportive culture and keeps experienced members invested.

#### Subtheme 3.3: Challenges at the Commitment Stage

Even in this advanced stage of engagement, participants encountered challenges that could dampen their participation or slow their progress in the community. This study identified participants’ selective engagement stemming from concern about being misinformed and misinforming others, a mismatch between information seeking and sharing, and limited incentive and structural support to transition between roles.

##### Selective Engagement Stemming From Concerns About Misinforming Others and Being Misinformed

Members were highly conscious of avoiding the spread of false information, which sometimes made them overly cautious about posting. Participant 7, for example, admitted that this worry makes them “very careful” about what they share, saying that “And you’re very careful when you're giving information.” Participant 2 also corroborated this point, noting that:

... o I was very cautious about what information I would share. I’m not an expert. So I really didn’t share ... I would share information that I’ve learned on the site with my physician, but not with the folks on the site

Many users prioritized credibility over frequency of posts, suggesting that misinformation concerns act as a filter for participation, with users engaging more selectively to uphold community trust. While such caution helps maintain trust in the community, it also leaves some users uncertain about contributing information, which means some useful contributions never get made. Participant 5, for instance, recalled hesitating to share a personal success story even after consulting her doctor, second-guessing whether to post it. They mentioned that:

*... Even though I checked with my doctor, I didn’t know if there were any downsides to it. But I thought No, that’s good advice, I should say that.*

[Participant 5]

In cases like Participant 5’s, valuable experiences might be withheld due to self-censorship, limiting the breadth of knowledge available. Overall, this extra cautiousness indicates that misinformation concerns can dampen overall engagement levels within OHCs. While this behavior may enhance the quality of shared information and trust, it may also create barriers to open participation if not addressed, as users self-censor or second-guess their contributions to avoid misinformation risks.

##### Limited Support for Role Transitions

Another critical finding that emerged in our study is the challenge of limited transition support, as members become more knowledgeable about their health and desire to take on more supportive roles. Participants who have had personal experiences managing CVD and gained self-management knowledge expressed having limited structural support and incentives for long-term use in transitioning from information seekers to more active information and emotional support providers. While a few participants, like Participant 7, received support that fostered their sense of belonging and propelled them to take on more supportive roles, some other participants reported not having the pathways to supporting others within the community. For example, Participant 2, who has joined the community for X years and supposedly learned a lot about their condition, said that:

People that have gone through CVD and now that they’re healthier or have a better understanding, where they could teach other people. I didn’t seem to find that on the site.

Most of the participants who have maintained active engagement within the community expressed interest in providing support and mentorship to newly diagnosed members. However, they reported that the platform is not structurally adaptive enough to foster participants’ transition to more active roles.

### Theme 4: Community Integration—Sustaining Engagement Long-Term and Forging Community Integration

#### Overview

In the community integration stage, members sustain their engagement and solidify their roles in the OHC, contributing to its stability and growth. They identified as regular, integral contributors who mentor others, advocate for the community, and help improve the platform’s functionality and reach. The perceived motivation and behavior in this stage include commitment stabilization through mentorship and community advocacy, and development, respectively.

#### Subtheme 4.1: Commitment Stabilization and Mentorship

Participants reported having more stable and supportive roles in the community. They served as mentors, peer leaders, and trusted supporters. In these roles, they regularly offered emotional support, guidance, and resources to newer members. By this stage, their engagement is driven less by personal information needs and more by a sense of responsibility, community identity, and reciprocity. These committed members became pivotal in sustaining a collaborative, welcoming atmosphere. Their consistent presence fostered trust and encouraged new members to remain active. For example, Participant 3 (an aphasia survivor) led a stroke peer group for 3 years and cocreated a new aphasia subgroup, reflecting long-term commitment to the community:

There’s a new one called Aphasia Peer Group. I’ve been the lead for stroke for those three years and also now have the Aphasia Peer Group and that’s all new.

As a mentor, Participant 3 prioritized finding useful resources for others, indicating a shift from personal needs to actively supporting peers:

I’m not there to look up information; what’s wrong that I could use for myself as much as I’ll be looking for resources so I can forward them on to other people within my group or to other groups and of course I’ll respond to the people since I’m the lead

This stable commitment marks their evolution into trusted supporters fully integrated in the community.

#### Subtheme 4.2: Community Advocacy and Development

In the integration stage, participants extended their efforts into community advocacy and platform development. Some actively promoted the community to others outside the platform who could benefit from it. Others recruited new members to broaden the community’s impact. They also provided feedback to improve the platform’s features and usability, demonstrating commitment to the community’s growth. For instance, Participant 3, who cocreated a new aphasia peer group, observed that the next step was encouraging more hesitant members to participate. They noted that:

Now getting the members to see that there are these peer groups and start to engage with the peer groups is probably the next step.

[Participant 3]

Leadership roles like this foster long-term engagement but also bring challenges, especially in engaging more risk-averse members. Participant 3 acknowledged how much effort is required to get such members actively involved:

Well, it’s been years to get to this point with the peer groups, with Aphasia Peer Group. It takes a long time just to get those people who are otherwise risk-averse to join members like this, groups like this. Now we have to get them to start asking questions, which is sort of the next step.

Participant 3’s reflection illustrates a dedication to the community’s longevity and growth. These behaviors show how users at the integration stage take on higher responsibilities that sustain the community’s core operations and foster its continued vitality and expansion.

### Opportunities for Design to Support Long-Term Engagement

#### Theme 1: Opportunity for Adaptive Recommendation Systems to Improve Stage-Based Personalization

Users in the discovery and exploration stages often face overwhelming amounts of information, which can impede engagement. Consequently, all participants expressed interest in automatically receiving recommendations based on their profiles for activities they might like best, such as articles to read, topics to discuss, groups to join, and people to connect with in the online community. For example, Participant 4 emphasized the importance of making it easier for users to locate tailored and personalized content within the platform, saying that:

... but I think the most important thing is that when somebody goes on this website, it should be much easier to find their way to something relevant.

Corroborating Participant 4, some participants also noted the limitations of existing broad subgroups. While the AHA support network platform provides condition-specific groups, some participants, such as Participant 10, sought finer-grained distinctions that align with unique personal circumstances and pointed out the need for recommendations based on specific causes of atrial fibrillation.

... they’ll say AFib, but AFib has a million causes. Mine was caused just by what the surgeon did to me, whatever. And so even in that sense, people say AFib, but there’s so many causes. I mean some people just get it and they don’t know why, so I don’t have anything in common with them.

[Participant 10]

This feedback suggests the need for finer-grained personalization. This finding highlights the opportunity to implement an intelligent system that anticipates users’ needs at different stages in their journey, could guide each user to the right resources, and help newcomers overcome information overload. The adaptive systems could predict users’ needs at different stages, prediagnosis, postdiagnosis, or postprocedure, and deliver targeted articles, groups, and connections that align with their health journeys.

#### Theme 2: Opportunity for Patient Education to Optimize Use Practices

Across all stages, a recurring theme was the need for better education about OHC use norms and practices. Many participants were cautious about sharing information due to concerns about misinformation or perceived inadequacy in their expertise. Despite possessing lived experiences in managing CVDs, they felt that their potential contributions were less substantial because they are not medical professionals. This perceived deficit in competence adversely affects their self-efficacy, deterring active participation and sharing potentially beneficial personal experiences.

In their suggestions for addressing this challenge, participants expressed that patient education and digital literacy intervention could help improve their self-efficacy and competence level to enhance optimal information exchange practices. For example, Participant 8 proposed incorporating educational interventions such as live webinars or interactive events to guide users in responsibly seeking and sharing information:

I don’t know if you can do a live event within the context of a support group, or you could do live events or even webinars and direct through the support group to there, but it seems to me that this is an avenue to do education from American Heart on issues which are clearly in need of explanation and education.

Educating users on how to both seek and share information would build their self-efficacy to participate, especially as they become more experienced. Providing structured resources (eg, interactive tutorials) can further prepare willing members to take on mentor or leader roles, while also bridging any health or technology literacy gaps that might hinder participation.

#### Theme 3: Facilitating and Supporting Role Transitions Within the Community

As members become more experienced, many are eager to give back by mentoring others or leading groups, but participants noted little guidance for taking on such volunteer roles. Participant 2, who discussed being primarily motivated to support other participants, expressed that while they were willing to take on more responsibilities, they often lacked the guidance or resources needed to perform these roles effectively, saying that:

People who have gone through CVD and are now that they’re healthier or have a better understanding, where they could teach other people. I didn’t seem to find that on the site.

This finding suggests a need for structured support to facilitate experienced users’ transitioning into supportive roles such as mentors, moderators, or peer group leaders effectively. Platform managers and designers can address this by defining specific volunteer roles and offering training to help experienced users step up. Providing clear role descriptions, structured onboarding, and resources like peer-led workshops or handbooks could prepare members for mentorship and leadership duties. By facilitating these transitions, the platform can better harness experienced members and help the community thrive.

#### Theme 4: Interest in Alternative Activities and Modes of Engagement

Participants in the commitment and integration stages expressed interest in exploring immersive and synchronous activities to maintain engagement outside of the typical question-and-answer interactions in the group. Some participants spoke about their interest in health technological tools that could be helpful for daily health and lifestyle management and may create a new need for the SNC. For example, Participant 12, suggesting integrating tools like trackers for health monitoring, said:

I thought it would be nice to have a little bonus tools in there. It’s the same thing, a tracker, when you have your test, your vision when you visit a doctor, And it helps me figure out my journey through how I’m doing, which I think it will help others if they have it ... I couldn’t find something like that. I created mine, which I didn’t mind, but it would’ve been nice just to have ...

Participants also noted that synchronous activities, such as Zoom sessions and face-to-face gatherings, could invigorate discussions and create stronger bonds among members. Participant 12 emphasized how real-time interactions could enhance the community experience, particularly for those who value interpersonal connection over asynchronous communication:

the virtual Zoom meetings are the ones that I go to. I find that much more helpful. I see pictures of these people talking, and we can have an exchange. It’s wonderful for support even if we’re dealing with some different issues, but just the human contact I think is very good.

Members anticipate more dynamic conversations and a stronger sense of connection by participating in these live exchanges, potentially leading to more robust support networks. Such synchronous formats offer immediacy and presence that asynchronous forums may lack, potentially invigorating the community with active participation and a more cohesive collective experience. Implementing additional features that users want could provide dynamic interactions, particularly appealing to users in the commitment and integration stages, fostering stronger connections and the continuous value of the community.

## Discussion

### Principal Findings

This study explored perceived engagement patterns, including user behaviors, motivation, and needs in a large OHC through a stage-based framework. The study highlighted 4 distinct, nonlinear yet progressive engagement stages: discovery (crisis-driven initial engagement), exploration (navigation and orientation), commitment (active engagement and information management), and integration (sustained engagement and mentorship). Key barriers included information architecture complexity, concerns about misinformation, limited support for role transitions, and decreased participation as health management improved. Participants identified opportunities through which OHCs could increase stage-based engagement, including adaptive recommendation systems, health information literacy programs, structured role transition support, and alternative engagement modalities, such as synchronous interactions and health tracking tools. We summarized this stage-based model through a visual representation in Figure 1.

**Figure 1.**
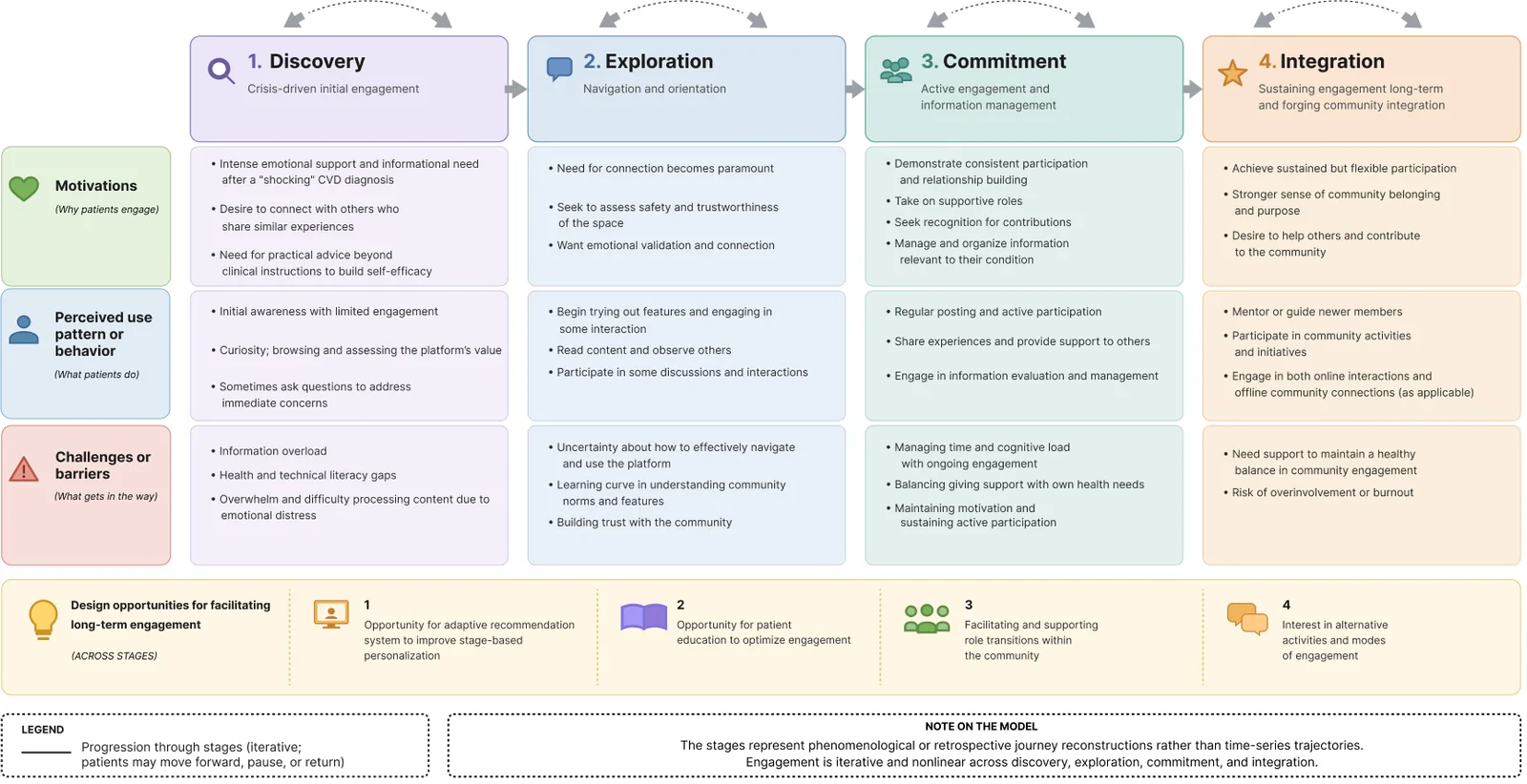
A stage-based model of perceived patients’ engagement in a large online health community. CVD: cardiovascular disease.

### Comparison With Prior Work

Previous research on OHC engagement has typically classified users into discrete categories such as lurkers, contributors, and leaders [40-42]. While these classifications provide useful snapshots of participation patterns, they obscure the dynamic nature of engagement over time. Our stage-based model extends this literature by revealing how and why users transition between different forms of participation. Rather than representing fixed user types, these patterns reflect different phases in members’ engagement journeys, with progression depending on whether the platform adequately supports their evolving needs.

Our findings align with legitimate peripheral participation theory [43], which posits that newcomers gradually move from peripheral observation to full community membership through increasing engagement and identity development. However, we found that this progression is neither automatic nor unidirectional. The discovery stage represents a critical intervention window where newly diagnosed patients seek information and emotional support during moments of crisis. Yet, many encounter barriers that prevent progression to deeper engagement. Consistent with existing studies [26,44,45], participants highlighted the overwhelming nature of navigating vast amounts of information during this stage. These barriers include information overload, health literacy gaps, and technical challenges that compound the emotional distress of a recent diagnosis. OHC architectures should account for the reduced cognitive capacity and heightened vulnerability of those in crisis-driven entry.

During the exploration stage, our findings challenge assumptions about how condition-specific communities should be structured. Participants sought connections not just with others sharing the same diagnosis, but with those sharing similar circumstances, treatment approaches, and demographic characteristics. This aligns with social identity theory [46], which emphasizes the importance of identity-based group formation for fostering belonging and sustained participation. The evolution from transactional information exchange to relational connection demonstrates how appropriate relationship formation enables shifts in engagement priorities and facilitates progression toward more active roles.

The commitment stage introduces a paradox not adequately addressed in prior OHC literature. As members become more knowledgeable and engaged, they develop sophisticated strategies for managing misinformation risks through disclaimer practices, systematic verification, and topic-based participation patterns. While these behaviors reflect responsible community norms, they also create barriers to the detailed, experience-based knowledge exchange that initially attracted members to the community. This tension between information seekers desiring specific, actionable details and information sharers providing cautious, generalized advice creates dissatisfaction on both sides and may contribute to reduced engagement over time.

A critical finding that extends existing engagement models is the fluid nature of user roles. Consistent with recent literature emphasizing the temporal dimensions of online participation [17,40,41], we found that users oscillate between information-seeking and information-sharing based on health status changes, knowledge saturation, and personal circumstances. What may appear as disengagement often represents appropriate adaptation to changing needs, such as members whose health has stabilized, maintaining peripheral connection through email alerts while reducing active participation within the community platform. This challenges linear models of engagement progression and suggests the need for platforms that accommodate fluid role transitions.

### Theoretical and Practical Implications

These findings have important theoretical and practical implications for understanding and supporting OHC engagement. Theoretically, our stage-based model advances understanding of how engagement evolves by specifying the mechanisms that drive progression and the barriers that impede it at each stage. This moves beyond static typologies to reveal engagement as a dynamic process shaped by the interaction between individual needs and platform affordances.

Several critical barriers emerged that prevent members from progressing through engagement stages. Information architecture complexity prevents users from locating relevant content during exploration, despite high initial motivation. Current platforms use broad categorization schemes that may not account for heterogeneity within diagnostic categories. Limited structural support for role transitions represents another critical gap. While participants expressed clear interest in providing mentorship as they gained experience, many lacked pathways to assume supportive roles. This reveals that role transition support requires more than opportunity; it requires structured onboarding, training, recognition mechanisms, and ongoing support from community managers.

From a practical standpoint, our findings suggest 4 design opportunities to address these barriers and support stage-based engagement. Adaptive personalization systems represent a promising approach to reducing overwhelming information volumes during discovery, facilitating identity-based connections during exploration, and identifying role transition opportunities during commitment and integration stages. Implementation should consider multiple dimensions, including diagnosis and cause, treatment approaches, demographic factors, health journey stage, and engagement preferences, leveraging both explicit user profiles and implicit behavioral signals.

Patient education and digital literacy support offer another opportunity for improvement. Educational interventions should address the perception that experiential knowledge is less valuable than medical expertise by clarifying appropriate boundaries between experiential sharing and medical advice, teaching effective verification strategies, normalizing disclaimer practices, and building confidence in peer support value. Such education should be stage-appropriate, from platform orientation during discovery to mentorship training during integration.

Addressing the challenge of role transitions requires structured support mechanisms that provide clear pathways from information-seeking to mentorship roles with transparent descriptions of responsibilities and available support. This includes formal onboarding processes, ongoing training opportunities, recognition systems, and community manager support. Given the fluid nature of user roles, platforms should support flexible transitions that accommodate members’ evolving needs while providing structure for those seeking to contribute.

Finally, diversifying engagement modalities through synchronous activities and health tracking tools can address unmet needs beyond asynchronous forums. Synchronous formats offer immediacy, social presence, and relationship-building opportunities particularly valuable during exploration and commitment stages, while hybrid approaches may best serve diverse member needs by combining asynchronous core functions with optional synchronous activities.

### Limitations

Several limitations should be acknowledged. First, our sample was drawn exclusively from the AHA SNC, which had over 12,000 users at the time of this study and focuses on CVD management. The AHA support network is a moderated forum hosted by a major health organization, and so, features like content moderation, the presence of expert material, or the specific community culture could influence engagement patterns in ways that might differ from other health foci (eg, unmoderated peer groups or those for other health conditions). While the availability of these features provided research depth, it may limit generalizability to other health conditions or OHC platforms with different structures, moderation approaches, or demographic compositions. Different disease communities may exhibit unique engagement patterns based on disease characteristics, prognosis, and treatment trajectories. As such, the proposed stage-based model should be viewed as a conceptual framework that may require adaptation across different community types. Second, our sample was predominantly White, highly educated, and older adults, which may not reflect the experiences of more diverse populations. Younger users, individuals from different racial and ethnic backgrounds, or those with lower educational attainment may face different barriers and have different engagement preferences. The older adult demographic may particularly influence our findings regarding digital literacy and platform navigation challenges. Moreover, our sample size (N=19), although appropriate for qualitative inquiry, may not fully capture the diversity of experiences within each engagement stage. Some stages may be underpinned by fewer cases, and additional research is needed to confirm the stability and completeness of these stages in broader or longitudinal studies. Third, our retrospective interview approach relied on participants’ recall of their engagement journey, which may be subject to memory biases or retrospective sense-making that does not accurately reflect their experiences at each stage. Additionally, the use of narrative analysis to construct stages of engagement may potentially limit our findings, as the data are based on retrospective self-reports that are vulnerable to post hoc rationalization and social desirability bias. Participants’ narratives may reflect how they currently make sense of past engagement rather than how engagement actually unfolded over time. As such, the staged model risks overinterpreting sense-making narratives as actual engagement trajectories. To ensure the validity of our claim, we present our findings as perceived engagement patterns. In the future, longitudinal studies following users from initial discovery through integration should be conducted to provide more accurate stage-specific data. Fourth, we did not collect systematic use data (eg, posting frequency, login patterns, and types of content engaged with) to triangulate with interview findings, which could have provided additional objective measures of engagement patterns. Finally, participants who agreed to be interviewed may represent more engaged or satisfied users, potentially missing perspectives of those who discontinued use or remained peripheral lurkers. This may bias the findings toward more affirming engagement trajectories and obscure potential barriers faced by disengaged or underserved users. Future work should aim to capture these critical viewpoints to enrich and challenge the current model.

### Conclusions

Through narrative thematic analysis, this study identifies perceived stage-based patterns in how participants retrospectively described their engagement with an OHC, suggesting that engagement is experienced as dynamic and nonlinear in relation to perceived changes in health status, knowledge, and social needs. By applying a stage-based framework—discovery, exploration, commitment, and integration—this study highlights how mechanisms such as self-efficacy building, trust formation, and role transition shape participation over time. While information needs drive initial engagement, sustained participation requires facilitators such as opportunities for meaningful contribution, relationship-building, and alternative forms of engagement beyond traditional question-and-answer interactions. Importantly, this study identified that concerns about misinformation not only affect information-seeking but also members’ willingness to share lived experiences, creating tension between safety and openness.

Sustaining engagement requires adaptive strategies that anticipate these shifts: personalized content delivery through machine learning, health information literacy programs to empower safe participation, and structured pathways for mentorship and leadership roles. Diversifying engagement modes, including synchronous interactions and self-management health tools, can further maintain the relevance and value of OHCs for experienced users. Designing for the full engagement cycle, from crisis-driven entry to integrated community leadership, offers a roadmap for platforms to foster resilience, reciprocity, and long-term value. Future research should validate these interventions across diverse communities and examine their impact on health outcomes. Ultimately, supporting continuity in engagement is essential for realizing the promise of OHCs as enduring sources of peer support and collective knowledge.

## Supplementary material

10.2196/90645 Multimedia Appendix 1 Qualitative interview guide.

10.2196/90645 Checklist 1 21-Item SRQR checklist for reporting qualitative study.

## Abbreviations

AHA: American Heart Association
SNC: Support Network Community
CVD: cardiovascular disease
OHC: online health community
