# Supplementary material for: Stage-Based Model of User Engagement Patterns in an Online Health Community for Cardiovascular Disease Management: Qualitative Interview Study
Source: J Med Internet Res. 2026 Oct 5;28:e90645. doi: 10.2196/90645 (PMC13638069; doi:10.2196/90645)
Supplement: Supplementary file 1 [file jmir-v28-e90645-s002.pdf]

21-item Standards for Reporting Qualitative Research (SRQR) adapted to report From Clicks to Community: A Qualitative Study of Stage-Based Engagement in an Online Health Community.

No	Topic	Item [Y = Included, N = Not Included]
**Title and Abstract**		
S1	Title	Y
S2	Abstract	Y
**Introduction**		
S3	Problem formulation	Y
S4	Purpose or research question	Y
**Methods**		
S5	Qualitative approach and research paradigm	Y
S6	Researcher characteristics and research reflexivity	Y
S7	Context	Y
S8	Sampling Strategy	Y
S9	Ethical issues pertaining to the human subjects	Y
S10	Data collection methods	Y
S11	Data collection instruments and technologies	Y
S12	Units of study	Y
S13	Data processing	Y
S14	Data analysis	Y
S15	Techniques to enhance trustworthiness	Y
**Results/Findings**		
S16	Synthesis and interpretation	Y
S17	Links to empirical data	Y [available upon request]
**Discussion**		

S18	Integration with prior work, implications, transferability, and contributions(s) to the field.	Y
S19	Limitations	Y
**Other**		
S20	Conflicts of interest	Y
S21	Funding	Y
