# Supplementary material for: Stage-Based Model of User Engagement Patterns in an Online Health Community for Cardiovascular Disease Management: Qualitative Interview Study
Source: J Med Internet Res. 2026 Oct 5;28:e90645. doi: 10.2196/90645 (PMC13638069; doi:10.2196/90645)
Supplement: Supplementary file 2 [file jmir-v28-e90645-s001.docx]

**Qualitative Interview Guide**

**Introduction**

Hello, my name is Monisola Jayeoba. I am a technology and social behavior doctoral researcher in the Computer Science and Communication Studies Department at Northwestern University. Thank you for agreeing to participate in an interview as part of the Support Network Community research. This support network research is in collaboration with the American Heart Association. We appreciate your willingness to share your thoughts with us about usage behaviors, benefits, and inadequacies of the support network community.

**General Objective**

The American Heart Association, in collaboration with researchers at Northwestern University, is interested in understanding your experiences with its online support network community. The overarching goal of this study is to understand the usage behavior and benefit of the support network community in order to explore aspects for improvement.

Through the data you will provide, we aim to develop and implement newer features to enhance or improve members’ experience with the community. Therefore, the purpose of our session today is to learn more about your preferences, ideas, and opinions regarding the American Heart Association Support Network Community.

**Guidelines**

a. There are no right or wrong answers. Please feel free to share your point of view. We are genuinely interested in your perspective.
b. Please keep in mind that we're just as interested in negative comments as positive comments, and at times the negative comments are the most helpful.
c. We will record today’s session because we don’t want to miss any of your comments. People often say very helpful things in these discussions, and we can't write fast enough to get them all down. Are you okay with this? [CLICK RECORD SESSION]
d. We are on a first-name basis. However, we will not use any names in our reports. If you do not want to use your real name because of the recording that is fine. Please let us know what you would like to be called or how you would like to be addressed.
e. Where necessary, I will use abbreviations like CVD to represent cardiovascular diseases and AHA to represent American Heart Association. Is that okay with you?
f. Do you have any questions before we start?

**Questions**

**Specific Aim -** Understanding health information-seeking behavior, motivation, and uncertainty in patient-to-patient communication*.*

***Information Seeking and Management.***

1. What year did you start using the online peer Support Network Community?
2. Could you share how you learned about Support Network Community and describe your reasons for joining and engaging in the community?
   1. Have these reasons for use changed over time since you first joined the community?
   2. Have these reasons been addressed within the community? Did you find the information or content you were looking for?
3. Could you describe how often you visit the community site?
4. When you visit the community site, what type of activities do you usually engage in? Examples of activities are browsing and reading posts and content, posting new questions, answering others’ questions, upvoting/liking content, and finding resources through topic forums or in peer groups, etc.
   1. How has your frequency of use changed over time i.e., when you joined and now?
   2. How do these changes relate to the status of your heart health?
   3. Were there instances where something changed in your medical or health condition that triggered more use of the online peer support network community? If yes, could you describe this more?
5. Many individuals learn about their conditions from various sources such as their healthcare provider and educational websites. Do you consider the online peer support community to be a source to learn about your heart and brain health? Why or why not?
6. What examples of posts have you found to be helpful to you and what are the reasons that you find them helpful?
   1. Can you describe an instance where you found information that you have applied towards managing or living with your condition)?
   2. Why did you decide to try it out?
   3. How did it turn out?
7. In addition to the AHA support network community, do you have other online sources/forums/communities where you get support or seek health information?
8. [If yes to 7] Why do you have multiple forums/groups/communities?
9. [If yes to 7] How do you balance multiple information sources? What determines which community you use and when you use it?
10. How do you assess the trustworthiness of content or posts in the community [if multiple communities, across multiple communities]?

**Misinformation, Risks and Uncertainties.**

*Uncertainty Definition: In this study, we adopt Brashers’s (2001) definition of uncertainty as existing “when details of situations are ambiguous, complex, unpredictable, or probabilistic; when information is unavailable or inconsistent, when people feel insecure in their own state of knowledge or the state of knowledge in general” (p.487)*

11. Have you made any engagement, such as posts or responses to others’ questions since joining the support network community?
12. [If No to 11], what do you think is causing you to have low engagement or communication levels in the community? [If Yes to 11], tell me when and what motivated you.
13. Within the online support network and even offline, is there any time you have shared content/information, for example, recommendations or answered questions but you were unsure or not very certain about the information you shared? Could you share details of [one of] the scenario(s)?
14. How and to what extent does your knowledge of misinformation impact your engagement in the online community, i.e how you communicate with other patients, caregivers, and health professionals in the community? Does the fear of misleading or misinforming others make you cautious or extra careful about how you communicate and engage in online communities?
15. Do you get nervous about sharing content or generally engaging in the community such as answering questions, providing recommendations, and posting about personal experiences? What kind of topics are you nervous about sharing or discussing?
16. Within AHA Support Network Community, do members talk about risks or disclose their level of uncertainty when they share information, for example, respond to questions or post recommendations to other members? If yes, please share an example that you recall or that stood out to you.
17. [If yes to 16] How did you perceive and process the risks or uncertainty in the health information? When people disclose risks and uncertainty levels with the information they share, how does that affect your attitude towards the information?
    1. When you make recommendations to other members or information seekers, and you disclose how uncertain you are or the risks involved in the details you share, does this promote positive attitude or negative towards the information?
18. Do members disclose the source(s) of the information they share, for example, based on their discussion with their clinicians, personal experiences, or opinion? If yes, in what context?
19. When you seek health information online for example, recommendations on a healthy diet or exercise routine or sleep pattern on AHA Support Network Community, would prefer that responders disclose [to the best of their abilities] what worked and did not work for them; and the risks of adopting the recommended idea? If yes, how would you want them to share or communicate it?
20. [If yes to 15] How might the risks or uncertainty information be relevant or beneficial to you and your health journey?
21. When you contribute health information online, for example, you provide recommendations on a healthy diet or exercise routine, or sleep pattern on AHA Support Network Community, do you disclose what has worked and did not work for you (or patient); and the risks that might be associated with adopting recommended product or process?
22. [If yes to 17], Could you please provide an example, perhaps your most recent one, and tell us what motivated you to disclose those risk and uncertainty details?
23. [If yes to 17], What reactions/responses/comments did you receive from other community members based on the post or information you shared?
24. What help could be provided to you to help you feel more confident about engaging in the community?
25. How can we help OR support you to understand misinformation and uncertainty such that they do not inhibit your contributions/communication/engagement on the AHA community.

**Recommendation system**

26. In the Support Network Community, do you find it easy or hard to find contents that are relevant to you and your health journey?
27. In the Support Network Community, do you find it easy or hard to find and connect with people who share similar experiences with you?
28. In what ways can the Support Network Community be better redesigned or improved to meet your needs?
29. Would you like to automatically receive recommendations based on your personal profile for what you might like best, such as articles to read, topics to discuss, interest groups to join, and people to connect with in the community?
30. [If yes to 29] What kind/type of recommendations would you want to receive?
31. Is there any question you have for me?

**Closing Remark**

Thank you for participating in this study. We appreciate your willingness to support cardiovascular health management research and hope that the research outputs will directly or indirectly benefit you and your loved ones.
